# Anticancer Activity of Amb4269951, a Choline Transporter-Like Protein 1 Inhibitor, in Human Glioma Cells

**DOI:** 10.3390/ph13050104

**Published:** 2020-05-25

**Authors:** Saiichiro Watanabe, Nozomi Nishijima, Kaho Hirai, Kaoru Shibata, Akane Hase, Tsuyoshi Yamanaka, Masato Inazu

**Affiliations:** 1 Institute of Medical Science, Tokyo Medical University, 6-1-1 Shinjuku, Shinjuku-ku, Tokyo 160-8402, Japan; saiichiro@gmail.com (S.W.); s22061193@stu.rakuno.ac.jp (N.N.); rulla.k67@gmail.com (K.H.); kaoruinoue08@gmail.com (K.S.); akaneko1123@icloud.com (A.H.); 2 Department of Molecular Preventive Medicine, Tokyo Medical University, 6-1-1 Shinjuku, Shinjuku-ku, Tokyo 160-8402, Japan; yamanaka@rtss.co.jp

**Keywords:** glioma, choline, transporter, choline transporter-like protein, ceramide, survivin

## Abstract

Choline transporter-like protein 1 (CTL1) is highly expressed in glioma cells, and inhibition of CTL1 function induces apoptotic cell death. Therefore, CTL1 is a potential target molecule for glioma therapy. Here, we investigated the therapeutic mechanism underlying the antitumor effects of Amb4269951, a recently discovered novel CTL1 inhibitor, in the human glioma cell line U251MG, and evaluated its in vivo effects in a mouse xenograft model. Amb4269951 inhibited choline uptake and cell viability and increased caspase-3/7 activity. CTL1-mediated choline uptake is associated with cell viability, and the functional inhibition of CTL1 by Amb4269951 may promote apoptotic cell death via ceramide-induced suppression of the expression of survivin, an apoptotic inhibitory factor. Finally, Amb4269951 demonstrated an antitumor effect in a mice xenograft model by significantly inhibiting tumor growth without any weight loss. Amb4269951 is the lead compound in the treatment of glioma and exhibits a novel therapeutic mechanism. These results may lead to the development of novel anticancer drugs targeting the choline transporter CTL1, which has a different mechanism of action than conventional anticancer drugs against gliomas.

## 1. Introduction

Primary brain tumors are very diverse, and the WHO classification categorizes them into approximately 150 types according to various characteristics. Gliomas are the most common type of malignant brain tumor, accounting for 25% of all cases. Although the occurrence of gliomas is rare, their prognosis is extremely poor, with the 5-year survival rate of glioblastoma (grade IV) being less than 10% [1,2]. Very few antitumor drugs are available for treating gliomas. Moreover, some cases are difficult to treat due to the emergence of resistance to the alkylating agent temozolomide, which has otherwise demonstrated effectiveness [3,4,5]. Since most of the other available anticancer agents for treating gliomas are also alkylating agents, there is a need for alternatives based on novel therapeutic mechanisms.

Choline is an organic cation that plays a critical role in the structure and function of biological membranes. Following uptake into the cell, choline is metabolized into molecules necessary for the body and is known to be involved in various physiological functions. There are three known metabolic pathways of choline, acetylation, phosphorylation, and oxidation, each of which metabolizes choline into substances utilized as part of physiological functions in the cell [6,7,8]. The intracellular transport system of choline is an important function of the rate-limiting step of the choline metabolic system, which is mediated by transporter molecules. The choline transporters currently known to transport choline are classified into three groups: high-affinity choline transporter 1 (CHT1), choline transporter-like proteins (CTL1-5), and low-affinity organic cation transporters (OCT1-2). Each transporter is characterized by different properties, including their affinity for choline, tissue distribution, sensitivity to the choline uptake inhibitor hemicolinium-3 (HC-3), sodium dependence, and substrate specificity [6,7,8].

In the clinic, choline positron emission tomography (PET) and choline PET-computed tomography are now commonly performed and have been very effective in imaging several tumor types, including brain tumors [9]. In a past study, ^18^F-choline PET showed a very high tumor/normal ratio in gliomas, with 10.5 in anaplastic astrocytoma and 13.2 in glioblastoma [10]. Therefore, the mechanism of choline accumulation in tumors is thought to involve choline uptake, which is increased by enhancement of the cell membrane synthesis system during active cell proliferation. The intracellular transport of choline is known to be mediated by the choline transporter, which has an important function as a rate-limiting step of the choline metabolic system and is a potential therapeutic target molecule for gliomas. Previous studies have shown that CTL1 is highly expressed in various types of cancer cells, and that inhibition of their choline uptake function induces apoptosis [11,12,13]. CTL1 is highly expressed in glioma cells, and its transporter-mediated uptake of choline is a target of various anticancer drugs, such as cisplatin, etoposide, vincristine, and temozolomide [14]. Therefore, CTL1 is likely to be a target molecule for glioma therapy.

In recent years, molecularly targeted therapeutics have been a mainstay of anticancer drugs. In this class of therapies, biopharmaceuticals based on monoclonal antibodies have made a significant contribution to cancer treatment. However, therapeutic agents for brain tumors such as gliomas must pass through the blood–brain barrier, and treatment with polymeric monoclonal antibodies were not indicated. Therefore, we used a plant-derived natural organic compound library to search for small-molecule compounds that inhibited CTL1-mediated choline uptake as well as cell viability, which led to the discovery of the isoquinoline derivative, Amb4269951 [15].

In this study, we investigated the therapeutic mechanism of the antitumor effects of Amb4269951 and evaluated its in vivo effects using a mouse xenograft model.

## 2. Results

### 2.1. Effect of Amb4269951 and Hemicholinium-3 (HC-3) on [^3^H]Choline Uptake

**Figure 1:**
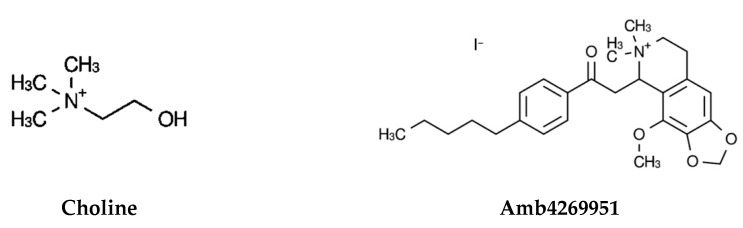
Chemical structure of choline (2-hydroxyethyl(trimethyl)ammonium) and Amb4269951 (4-methoxy-6,6-dimethyl-5-(2-oxo-2-(4-pentylphenyl)ethyl)-5,6,7,8-tetrahydro-[1,3]dioxolo [4,5-g]isoquinolin-6-ium iodide).

**Figure 2:**
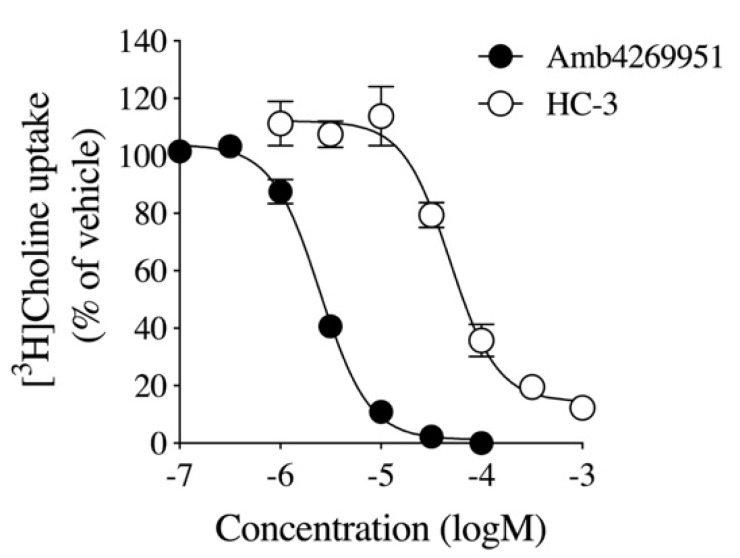
The effect of Amb4269951 and HC-3 on [^3^H]choline uptake in U251MG cells. Cells were pre-incubated with various concentrations of Amb4269951 and HC-3 for 20 min, and the uptake of 10 μM [3H]choline was then measured for 20 min. The results are given as a percentage of the control uptake measured in the presence of the vehicle. Each point represents the mean ± SD (*n* = 3). The IC_50_ values of Amb4269951 and HC-3 for the inhibition of [^3^H]choline uptake were 2.5 and 48.6 µM, respectively.

We examined the effects of Amb4269951 (Figure 1) and the choline uptake inhibitor, HC-3, on [^3^H]choline uptake in U251MG cells (Figure 2). HC-3 is a positive control, and its effect in inhibiting CTL1-mediated choline uptake in cancer cells was previously reported [11,12,13,14,16]. Both Amb4269951 and HC-3 inhibited choline uptake in a concentration-dependent manner, with IC_50_ values of 2.5 and 48.6 µM, respectively.

### 2.2. Effects of Amb4269951 on Cell Viability and Caspase-3/7 Activity

**Figure 3:**
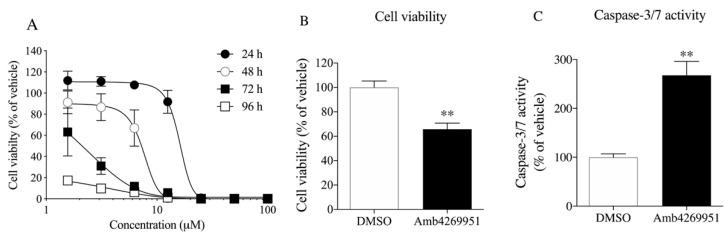
Effects of Amb4269951 on cell viability and caspase-3/7 activity in U251MG cells. (**A**) Cells were pre-incubated with various concentrations of Amb4269951 for 24, 48, 72, and 96 h, and then the cells were counted. The results are given as a percentage of the findings in the vehicle control. Each point represents the mean ± SD (*n* = 3). The effects of 6.25 µM Amb4269951 on cell viability (**B**) and caspase-3/7 activity (**C**) in U251MG cells. The cells were pre-incubated with 6.25 µM Amb4269951 for 24 h, and then the cell number and caspase-3/7 activity were measured. The results are given as a percentage of the findings in the vehicle control (DMSO). Each column represents the mean ± SD (*n* = 4). ** *p* < 0.01 compared to the vehicle control (DMSO).

We examined the concentration- and time-dependent effects of Amb4269951 on U251MG cell viability (Figure 3A). Amb4269951 inhibited cell survival in a concentration-dependent manner, and its effect was potentiated in a time-dependent manner. Amb4269951 significantly inhibited cell viability and significantly increased caspase-3/7 activity in U251MG cells (Figure 3B,C). We further investigated the effects of Amb4269951 on cell survival in various cancer cell lines. Amb4269951 suppressed cell survival of various cancer cell lines in a concentration-dependent manner (Figure S1).

### 2.3. Effect of Amb4269951 on the Expression of Sphingomyelinases

**Figure 4:**
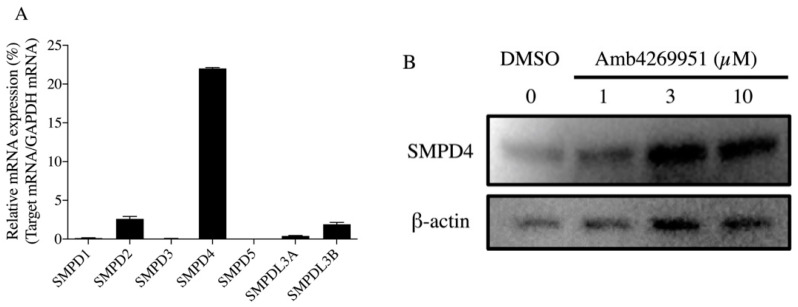
Effect of Amb4269951 on the expression of sphingomyelinases in U251MG cells. (**A**) Real-time PCR analysis of the mRNA expression of SMPD1-5, SMPDL3A, and SMPDL3B in U251MG cells. The percentage of relative mRNA expression is expressed as the ratio of the target mRNA to the glyceraldehyde-3-phosphate dehydrogenase (GAPDH) mRNA. Each column represents the mean ± SD (*n* = 3). (**B**) Effect of Amb4269951 on the expression of SMPD4 protein in U251MG cells. Cells were incubated with various concentrations of Amb4269951 for 4 h. Western blot analysis shows the SMPD4 and ß-actin levels in whole cell lysates of drug-treated U251MG cells.

The inhibition of choline uptake by choline uptake inhibitors is accompanied by a decrease in intracellular choline and a decrease in the content of phosphatidylcholine (PC), a major component of cell membranes. Cancer cells activate sphingomyelinases, hydrolytic enzymes of sphingomyelin, to increase the production of PC and ceramide, an apoptosis-inducing factor [17]. Therefore, the effect of Amb4269951 on the expression of sphingomyelinases was investigated. Since isoforms of sphingomyelinase have been reported [18], we first examined the mRNA expression of the various sphingomyelinase isoforms (SMPD1-5, SMPDL3A, and SMPDL3B) which are involved in ceramide production in U251MG cells using quantitative polymerase chain reaction (qPCR). U251MG cells predominantly expressed high levels of SMPD4 (Figure 4A). Treatment with Amb4269951 for 4 h resulted in a concentration-dependent increase in the protein expression of SMPD4 (Figure 4B).

### 2.4. Effects of Ceramide on Cell Viability and Caspase-3/7 Activity

**Figure 5:**
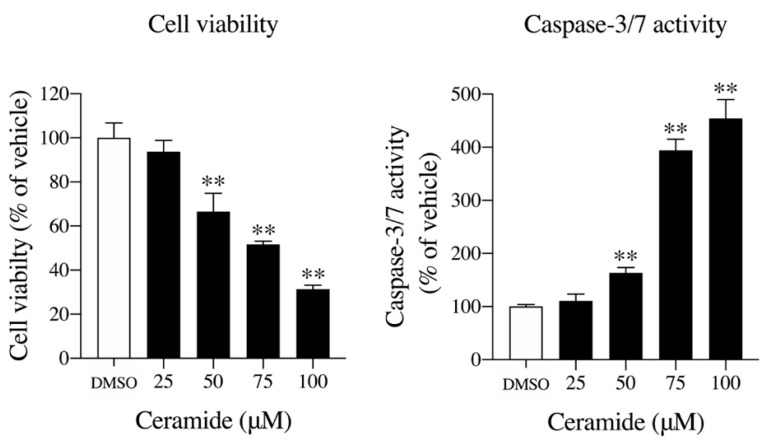
The effects of ceramide on cell viability and caspase-3/7 activity in U251MG cells. Cells were pre-incubated with various concentrations of ceramide for 12 h, and then the cell number and caspase-3/7 activity were measured. The results are given as a percentage of the findings in the vehicle control (DMSO). Each column represents the mean ± SD (*n* = 4). ** *p* < 0.01 compared to the vehicle control (DMSO).

The inhibition of choline uptake may trigger inhibition of the Kennedy pathway, which enhances the metabolic system of sphingomyelin to produce ceramide. Ceramide is known to be an apoptosis inducer, and increased ceramide production in the cell may induce cell death [19,20]. Therefore, we investigated the effect of ceramide on U251MG cells. After 12 h of ceramide treatment, cell viability decreased significantly in a concentration-dependent manner, and caspase-3/7 activity increased significantly in a concentration-dependent manner (Figure 5).

### 2.5. Effect of Amb4269951 and Ceramide on the Expression of Survivin

Survivin is the smallest member of the inhibitor of apoptosis family of proteins that are involved in the inhibition of apoptosis and regulation of the cell cycle. The overexpression of survivin promotes tumor progression in multiple pathways, including the dysregulation of apoptosis and cell division, altered sensitivity to anticancer drugs, and the promotion of cancer stem cell survival [21]. Thus, survivin is a target molecule in cancer therapy, and its inhibitors are potential therapeutic agents.

**Figure 6:**
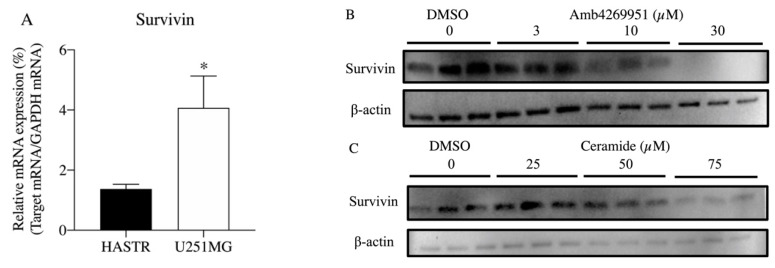
Effect of Amb4269951 and ceramide on the mRNA expression of survivin in U251MG cells. (**A**) Real-time PCR analysis of the mRNA expression of survivin in HASTR/ci35 and U251MG cells. The percentage of relative mRNA expression is expressed as the ratio of the target mRNA to the GAPDH mRNA. Each column represents the mean ± SD (*n* = 3). * *p* < 0.05 compared to the HASTR. (**B**) Effect of Amb4269951 on the expression of survivin in U251MG cells. Cells were incubated with various concentrations of Amb4269951 for 24 h. Western blot analysis shows survivin and ß-actin levels in whole cell lysates of drug-treated U251MG cells. The experiment was performed in triplicate. (**C**) The effect of ceramide on the expression of survivin in U251MG cells. Cells were incubated with various concentrations of Amb4269951 for 24 h. Western blot analysis shows survivin and ß-actin levels in whole cell lysates of drug-treated U251MG cells. The experiment was performed in triplicate.

Therefore, we examined the effect of Amb4269951 and ceramide on the expression of survivin, which is known to be highly expressed in tumor cells. The qPCR results revealed a significant increase in the expression of survivin in U251MG cells, compared to HASTR/ci35 cells (Figure 6A). Western blot analysis showed that 24 h treatment with Amb4269951 reduced the expression of survivin in a concentration-dependent manner (Figure 6B). The increase in SMPD4 expression due to Amb4269951 may induce apoptosis by degrading sphingomyelin and increasing ceramide production. Therefore, using ceramide, we also investigated whether survivin was involved in the induction of apoptosis. Treatment with ceramide for 24 h decreased the expression of survivin in a concentration-dependent manner (Figure 6B).

### 2.6. Effects of Amb4269951 in U251MG Cell Xenograft Model Mice

**Figure 7:**
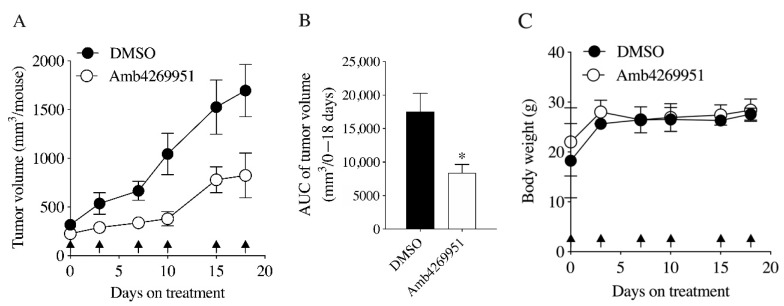
Effect of Amb4269951 on U251MG xenograft tumor progression and body weight. (**A**) The tumor volume was measured after intraperitoneal administration of 10 mg/kg Amb4269951 (*n* = 5) and the vehicle control with DMSO (*n* = 5). The date of administration is indicated by an arrow. (**B**) The Amb4269951 group significantly reduced xenograft tumor growth compared to the DMSO group (* *p* < 0.05). (**C**) The body weight of mice was measured at the time of drug administration. Data are represented as the mean ± SD (*n* = 5).

To evaluate the in vivo effects of Amb4269951, mice in a U251MG cell xenograft model were treated intraperitoneally with Amb4269951 (10 mg/kg body weight). As a result, the increase in tumor volume was significantly inhibited (Figure 7A,B) and no weight loss was observed, compared to the control group (Figure 7C).

## 3. Discussion

Increased choline uptake was reported to increase intracellular phosphocholine (PCho) levels in cancer cells [22,23,24]. PCho is a precursor of PC, a major component of the cell membrane that is essential for cell membrane synthesis. The transport of choline into the cell is an important function and the rate-limiting step of the choline metabolic system, and is mediated by the choline transporter. CTL1-mediated choline uptake is enhanced in cancer cells, and choline uptake may promote the synthesis of PC and sphingomyelin, which are major components of cell membranes and may be used for cell proliferation. Therefore, CTL1 is a novel target molecule for cancer therapy.

There are very few antitumor drugs for gliomas, among which temozolomide, an alkylating agent, contributes to the treatment of malignant gliomas. However, a cure is not possible in some cases, due to the emergence of drug resistance. Effects on the expression of the DNA repair enzyme O6-methyl-guanine-DNA-methyltransferase (MGMT) have been suggested as a cause underlying the development of drug resistance [25]. The protein expression of MGMT in tumor cells is epigenetically regulated, and a reduction in MGMT expression due to methylation of the MGMT promoter region precludes MGMT from carrying out its function in DNA repair, resulting in cells being highly sensitive to temozolomide. By contrast, patients in which the promoter region of MGMT is not methylated are able to express MGMT, resulting in the rapid DNA repair and resistance to alkylating agents [4]. Since most anticancer agents against glioma are alkylating agents, there is a need for alternative anticancer agents employing novel therapeutic mechanisms against glioma.

In this study, we investigated the mechanism of apoptotic cell death induction by the isoquinoline derivative, Amb4269951, which was previously discovered from a library of plant-derived organic compounds during our search for small-molecule compounds that inhibit CTL1-mediated choline uptake as well as cell viability [15].

Firstly, the inhibitory effect of Amb4269951 was compared with that of the choline uptake inhibitor, HC-3. Both Amb4269951 and HC-3 inhibited choline uptake in U251MG cells with IC_50_ values of 2.5 and 48.6 µM, respectively. The inhibitory effect of Amb4269951 on choline uptake was about 20 times stronger than that of HC-3. Choline is a cation under physiological conditions, and the function of CTL1 is competitively inhibited by a variety of organic cations [7,11,12,26]. The cationic site of choline is trimethyl, while that of Amb4269951 is dimethyl, and may serve as the recognition site of CTL1. In future, the structure–activity relationship should be clarified by comparing the compounds that modify the cationic moiety of Amb4269951. The CTL1-mediated inhibition of choline uptake in U251MG cells was also reported in anticancer drugs such as cisplatin, etoposide, vincristine, and temozolomide [14]. CTL1 inhibition may be involved in the antitumor effects of existing anticancer drugs. Many studies reported that various organic cations inhibit choline uptake and cell viability. Moreover, a strong correlation was observed between the inhibitory effect of organic cations on choline uptake and cell viability [7,11,12]. We found that Amb4269951 inhibited cell viability and enhanced caspase-3/7 activity in U251MG cells. In addition, the inhibitory effect of Amb4269951 on cell viability was potentiated in a time-dependent manner. The time-dependent suppression of cell viability may be the result of Amb4269951 inhibiting choline uptake and suppressing the choline metabolic system involved in cell survival.

The inhibition of choline uptake by choline uptake inhibitors leads to a decrease in PCho and PC content due to a decrease in intracellular choline. As a compensatory response to prevent the reduction of PCho and PC, cancer cells hydrolyze sphingomyelin through the action of sphingomyelinases, which promotes the production of PC and ceramide, an apoptosis-inducing factor. As a result, ceramide induces apoptotic cell death [7,27,28]. We examined whether the antitumor effects of Amb4269951 were mediated by these mechanisms. Amb4269951 increased the expression of SMPD4, which is mainly expressed in U251MG cells, in a concentration-dependent manner. These results suggest that SMPD4 hydrolyzes sphingomyelin to produce PC and ceramide. To investigate the effects of ceramide on cell proliferation and caspase activity, we found that ceramide inhibited cell viability in a concentration-dependent manner and simultaneously enhanced caspase-3/7 activity in U251MG cells. These results suggest that Amb4269951 may exert its antitumor activity through a ceramide-mediated apoptosis-inducing mechanism. In future, we must verify that Amb4269951 treatment enhances ceramide production in U251MG cells.

The anticancer effects of ceramide were reported in many types of cancer, and it was also reported to inhibit the expression of survivin, an apoptosis inhibitor, as well as enhance caspase activity, causing cell death [29,30]. We therefore focused on survivin, which acts as an endogenous inhibitor of the apoptotic enzyme caspase. Survivin is highly expressed in human cancers, though rarely detected in most normal tissues [31]. We found high levels of survivin expression in U251MG cells compared to normal astrocytes. It was suggested that higher survivin expression was associated with worse overall survival in patients with glioma [32]. Recently, survivin has attracted attention as a therapeutic target molecule for glioma [33,34,35]. We found that Amb4269951 and ceramide inhibited the expression of survivin in a concentration-dependent manner. These results indicate that Amb4269951 inhibits CTL1-mediated choline uptake, thereby activating SMPD4 and inducing ceramide production. Thus, ceramide may inhibit the expression of survivin and increase its downstream caspase activity to induce apoptosis-induced cell death.

Finally, the application of Amb4269951 in a mice xenograft model led to significantly inhibited tumor growth without any weight loss. Existing anticancer drugs are known to cause weight loss, suggesting that Amb4269951 may be a relatively safe anticancer drug, though further investigations on Amb4269951 would be needed to determine this.

These results may potentially lead to the development of novel anticancer drugs targeting components of the choline metabolism, including the choline transporter CTL1, which has a different mechanism of action than the conventional anticancer drugs against gliomas. Amb4269951, an isoquinoline derivative discovered in a library of plant-derived organic compounds, is the lead compound in the treatment of glioma with a novel therapeutic mechanism. Choline PET has shown to be highly effective in the imaging of several types of tumors, including prostate cancer, breast cancer, lung metastases from esophageal cancer, and mediastinal lymph node metastases. Hence, this therapeutic mechanism may also be useful for treating other tumors, in addition to gliomas.

## 4. Materials and Methods

### 4.1. Cell Culture

The human glioma cell line, U251MG, was purchased from the Japanese Collection of Research Bioresources Cell Bank (Osaka, Japan). U-251MG cells were grown in Dulbecco’s modified Eagle’s medium (D-MEM; FUJIFILM Wako Pure Chemical Corporation, Osaka, Japan) supplemented with 10% fetal bovine serum (Biowest SAS, Nuaillé, France) and penicillin (100 units/mL) streptomycin (100 µg/mL) solution (FUJIFILM Wako Pure Chemical Corporation, Osaka, Japan) in non-coated flasks. Cultures were maintained in a humidified atmosphere of 5% CO_2_ and 95% air at 37 °C, and the medium was changed every 2–3 days.

Immortalized human normal astrocytes (HASTR/ci35) were kindly provided by Dr. Tomomi Furihata of Chiba University [36]. HASTR/ci35 cells were routinely grown at 33 °C with 5% CO_2_/95% air in Gibco^®^ Astrocyte Medium (A1261301, Life Technologies, Carlsbad, CA, USA) supplemented with 1% N2 supplement, 10% fetal bovine serum (FBS), penicillin–streptomycin, and 4 µg/mL blasticidin S (SantaCruz Biotechnology, Inc., Dallas, TX, USA). For HASTR/ci35 cell differentiation, a culture medium without FBS was used, and the culture temperature was set at 37 °C.

### 4.2. Isoquinoline Derivative Amb4269951

Amb4269951 (4-methoxy-6,6-dimethyl-5-(2-oxo-2-(4-pentylphenyl)ethyl)-5,6,7,8-tetrahydro-[1,3]dioxolo [4,5-g]isoquinolin-6-ium iodide) was purchased from Greenpharma SAS (Orléans, France). Amb4269951 was dissolved in DMSO and used in the experiments. DMSO was used as a solvent control, and the final concentration was set at 1%.

### 4.3. [^3^H]Choline Uptake in U251MG Cells

U251MG cells were seeded into 24-well plates at 1 *×* 10^5^ cells/well. The [^3^H]choline uptake assay was performed 24 h later, after first discarding the medium, then washing the cells with uptake buffer (125 mM NaCl, 4.8 mM KCl, 1.2 mM CaCl2, 1.2 mM KH2PO4, 5.6 mM glucose, 1.2 mM MgSO4, and 25 mM HEPES, pH 7.4). After the addition of uptake buffer, [^3^H]choline was added to a final concentration of 10 µM, and uptake was initiated. Following [^3^H]choline uptake, the uptake buffer was aspirated, and cells were washed twice with pre-chilled uptake buffer. The cells were lysed in 0.1 M NaOH, 0.1% Triton X-100 solution, and the amount of [^3^H]choline taken up into the cells was measured using a liquid scintillation counter (Tri-Carb1 2100 TR, Packard Instrument Company, Meriden, CT, USA).

### 4.4. Cell Viability Assay

Cells were plated and cultured on 24-well plates. Amb4269951 and ceramide (C2 ceramide, Cayman Chemical, Ann Arbor, MI, USA) were added, and the final volume of medium in each well was 1.0 mL. C2 ceramide is a biologically active, cell permeable, and less hydrophobic analog of natural ceramides. Cell counts were measured using CellTiter-Glo^®^ Luminescent Cell Viability Assay (Promega, Madison, WI, USA) according to the manufacturer’s instructions. Luminescence was measured on a FilterMax F5 Multi-Mode Microplate Reader (Molecular Devices, LLC, Sunnyvale, CA, USA).

### 4.5. Measurements of Caspase-3/7 Activity

Caspase-3/7 activity, an indicator of apoptosis, was measured by a luminescence assay using caspase-Glo^®^ 3/7 Assay Systems kit (Promega, Madison, WI, USA). This kit is based on the cleavage of the DEVD (Asp-Glu-Val-Asp) sequence of a luminogenic substrate by caspase-3/7, which results in a luminescent signal. The cells were plated and cultured on 24-well plates. Amb4269951 and ceramide were added, and the final volume of medium in each well was 1.0 mL. Caspase-3/7 activity and live cell counts were measured using the caspase-Glo^®^ 3/7 Assay Systems and CellTiter-Glo^®^ Luminescent Cell Viability Assay, respectively. Caspase-3/7 activity was assessed as the unit of activity per viable cell by simultaneously measuring the number of viable cells. Luminescence was measured on a FilterMax F5 Multi-Mode Microplate Reader.

### 4.6. Quantification of mRNA by qPCR

The total RNA of U251MG and HASTR/ci35 cells was extracted using a QIAshredder and an RNeasy^®^ Mini Kit (Qiagen, Venlo, Netherlands) according to the manufacturer’s instructions. The pairs of primers and the TaqMan probes for the target mRNAs were designed based on the human mRNA sequence using TaqMan^®^ Gene Expression Assays (Assay ID: sphingomyelinase 1 (SMPD1), Hs03679346_g1; SMPD2, Hs00906924_g1; SMPD3, Hs00920354_m1; SMPD4, Hs04187047_g1; SMPD5, Hs04994298_g1; SMPDL3A, Hs01041066_m1; SMPDL3B, Hs01038741_m1; survivin (also known as BIRC5), Hs00977612_mH; GAPDH, Hs99999905_mL, Applied Biosystems, Foster City, CA, USA). For one-step real-time PCR, 20 ng of total RNA was added to the master mix of the TaqMan^®^ RNA-to-CT^TM^ 1-Step Kit (Applied Biosystems, Foster City, CA, USA) according to the manufacturer’s instructions. The RNA was then analyzed using the LightCycler^®^ 96 system (Roche Diagnostics, Mannheim, Germany). The relative mRNA expression levels of the target genes in cells were calculated using the comparative threshold cycle (Ct) method, according to previous studies [11,12]. The mRNA expression level relative to GAPDH for each target PCR was calculated using the following equation: relative mRNA expression = 2^−(Ct target − Ct GAPDH)^ × 100% [11,12].

### 4.7. Western Blot Analysis

Protein extraction from the cells was performed according to a previous method, as described below [11,12,13]. Cells in a Radio Immunoprecipitation Assay Lysis Buffer containing 1 mM ethylenediaminetetraacetic acid and protease inhibitors were extracted on ice by ultrasonic disruption followed by centrifugation (14,000g) for 15 min at 4 °C. The supernatant was incubated for 5 min at 100 °C in a 1:1 (v/v) ratio of Tris-SDS β-ME sample solution and electrophoresed on a 10% Mini-PROTEAN^®^ TGX^TM^ Gel (BioRad Laboratories, Inc., Hercules, CA, USA) along with a molecular weight standard. Proteins separated on 10% SDS-PAGE were transferred to polyvinylidene fluoride membranes using the Trans-Blot^®^ Turbo^TM^ Transfer System (BioRad Laboratories, Inc.). After protein transfer, the membrane was blocked with iBind^TM^ Flex Solution (Thermo Fisher Scientific Inc., Waltham, MA, USA) overnight at 4 °C. The membranes were then incubated with 4 µg/mL anti-SMPD4 antibody (ab133935, Abcam plc, Cambridge, UK) and 4 µg/mL anti-survivin antibody (ab76424, Abcam plc, Cambridge, UK) in an iBind^TM^ Flex Solution overnight at 4 °C. The membranes were then washed three times in iBind^TM^ Flex Solution and incubated with 1 µg/mL horseradish peroxidase-conjugated anti-rabbit IgG (Kirkegaard and Perry Laboratories Inc., Gaithersburg, MD, USA) and 1:1000 anti-β-actin pAb-HRP-DirecT (PM053-7, Medical & Biological Laboratories Co., Ltd., Nagoya, Japan) for 1 h at room temperature. The membranes were washed with iBind^TM^ Flex Solution, and protein bands were visualized using ECL^TM^ Prime Western Blotting Detection System (GE Healthcare Life Sciences, Buckinghamshire, UK). Luminescent images were obtained using a ChemiDoc XRS+ System (Bio-Rad Laboratories, Hercules, CA, USA).

### 4.8. Mouse Xenograft Model

All animal experiments were performed according to the protocol approved by the Animal Research Committee of Tokyo Medical University. A suspension of U251MG cells (1 *×* 10^7^ cell/mice) was subcutaneously injected into the dorsal part of 6-week-old male nude mice (BALB/cAJcl-nu/nu, CLEA Japan, Inc., Shizuoka, Japan). There were 5 per cage under pathogen-free conditions. The solid tumors which developed were then transplanted into the following 6-week-old male nude mice as 3 mm square blocks. Body weight as well as the long diameter (L) and short diameter (W) of the tumor were measured, and the tumor volume was estimated as L × W2 × 1/2. When the tumor volume reached approximately 100–300 mm^3^, Amb4269951 was administered intraperitoneally at a dose of 10 mg/kg, with 100% DMSO used in the control. Amb4269951 solution and 100% DMSO (100 μL/mice) were administered intraperitoneally on days 0, 3, 7, 10, 15, and 18. The mice were humanely killed when the tumor volume exceeded 2000 mm^3^.

## Supplementary Materials

The following are available online at https://www.mdpi.com/1424-8247/13/5/104/s1, Figure S1: Effect of Amb4269951 on cell viability in various cancer cell lines. Cells were pre-incubated with various concentrations of Amb4269951 for 24 h, and then the cells were counted. The results are given as a percentage of the findings in the vehicle control. Each point represents the mean ± SD (*n* = 4).
