# Supplementary material for: Anticancer Activity of Amb4269951, a Choline Transporter-Like Protein 1 Inhibitor, in Human Glioma Cells
Source: Pharmaceuticals (Basel). 2020 May 25;13(5):104. doi: 10.3390/ph13050104 (PMC7281368; doi:10.3390/ph13050104)
Supplement: Supplementary file 1 [file pharmaceuticals-13-00104-s001.pdf]

Supplementary Materials

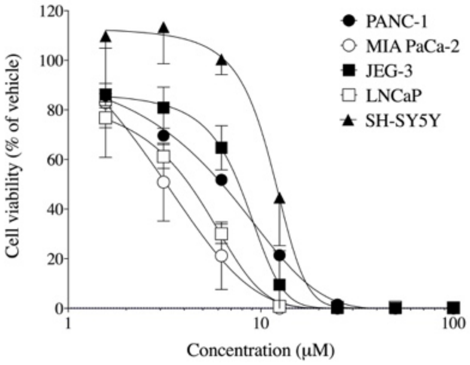

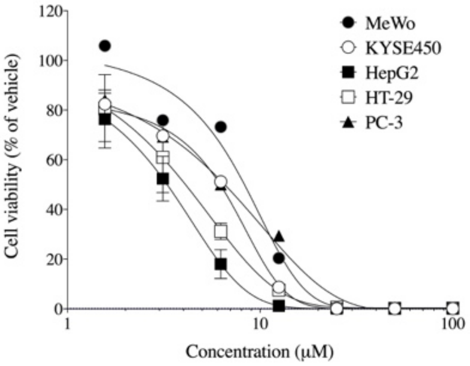

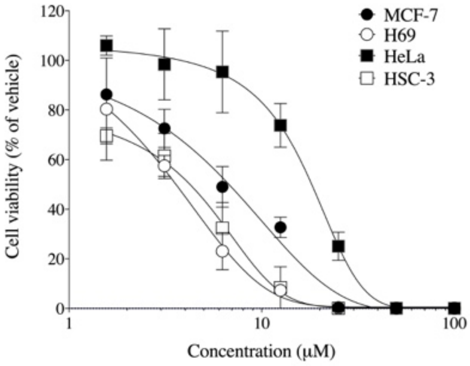

PANC-1	Pancreatic carcinoma cells
MIA PaCa-2	Pancreatic carcinoma cells
JEG-3	Choriocarcinoma
LNCaP	Androgen-sensitive prostate cancer cells
SH-SY5Y	Neuroblastoma
MeWo	Melanoma
KYSE-450	Esophageal cancer cells
HepG2	Hepatocellular carcinoma
HT-29	Colon adenocarcinoma
PC-3	Androgen-insensitive prostate cancer cells
MCF-7	Breast cancer cells
H69	Small cell lung carcinoma
HeLa	Cervix adenocarcinoma
HSC-3	Oral squamous carcinoma

Figure S1: Effect of Amb4269951 on cell viability in various cancer cell lines. Cells were pre-incubated with various concentrations of Amb4269951 for 24 h, and then the cells were counted. The results are given as a percentage of the findings in the vehicle control. Each point represents the mean ± S.D. (*n* = 4).
